# Kidney diseases and single-cell sequencing research: a bibliometric analysis from 2015 to 2024

**DOI:** 10.1080/0886022X.2025.2521457

**Published:** 2025-06-23

**Authors:** Yaotan Li, Jinyi Hou, Xinghua Zhang, Xiaochang Wu, Shijia Lin, Weijing Liu, Yaoxian Wang, Huijuan Zheng

**Affiliations:** ^a^ Dongzhimen Hospital, Affiliated to Beijing University of Chinese Medicine, Beijing, China; ^b^ Institute of Nephrology, Beijing University of Chinese Medicine, Beijing, China; ^c^ Henan University of Chinese Medicine, Zhengzhou, China

**Keywords:** Single-cell RNA sequencing, kidney diseases, cellular heterogeneity, bibliometric analysis, research trends

## Abstract

**Background:**

Single-cell RNA sequencing (scRNA-seq) has revolutionized kidney disease research by enabling high-resolution transcriptomic analysis at the cellular level. This technology can overcome the limitations of traditional bulk-sequencing; reveal disease-progression trajectories, intercellular communication networks, and cellular heterogeneity; and provide crucial insights into disease mechanisms, thereby facilitating the development of targeted therapies and personalized treatment strategies. We conducted a bibliometric analysis of publications describing the use of scRNA-seq in kidney disease research from 2015 to 2024 using the Web of Science Core Collection (WoSCC) database. Data analysis was performed using the R packages Bibliometrix, VOSviewer, and CiteSpace to systematically evaluate the research landscape and emerging trends.

**Results:**

A total of 1,210 publications on scRNA-seq in kidney diseases were identified. China was the largest contributor among the participating countries, demonstrating consistent annual growth in publication numbers. The major research institutions were Harvard Medical School, Sun Yat-sen University, and Shanghai Jiao Tong University. Most articles in this field were published by *Frontiers in Immunology*. In a list of 8,984 authors, the most productive authors were B. D. Humphreys, Haojia Wu, and Matthias Kretzler. The dominant categories identified in this search were scRNA-seq, disease progression/mechanisms, and gene regulation/expression. Several budding areas of investigation were also noted, including immunotherapy and scRNA-seq innovations, which allude to active evolution in the field.

**Conclusion:**

This bibliometric analysis revealed the rapid growth and evolving landscape of scRNA-seq applications in kidney disease research and highlighted promising opportunities for understanding disease mechanisms and developing personalized therapeutic strategies.

## Introduction

Single-cell RNA sequencing (scRNA-seq) has emerged as a powerful tool for kidney disease research. The fundamental principle of scRNA-seq involves isolating individual cells, extracting and reverse-transcribing RNA to complementary DNA (cDNA), amplifying the cDNA, constructing sequencing libraries, and performing high-throughput sequencing followed by computational analysis. The approach has substantially advanced our knowledge of the diversity of cell types in complex biological systems [1]. Moreover, this strategy offers several notable benefits over traditional bulk-sequencing approaches: the ability to perform unbiased high-throughput profiling at single-cell resolution circumvents the averaging bias inherent in population-level investigations, since traditional bulk RNA sequencing of kidney tissues in diabetic kideny disease (DKD) failed to distinguish the differential responses of podocytes, mesangial cells, and endothelial cells in the glomerulus, leading to an oversimplified understanding of disease mechanisms. Additionally, this technology has been shown to improve the detection sensitivity of smaller cell subpopulations that may be masked in aggregated sequencing data, and the platform’s compatibility with multi-omics workflows allows integrative investigation of cellular behaviors across various molecular dimensions [2,3]. Therefore, the application of scRNA-seq in nephrology deserves particular attention because of the kidney’s unique cellular architecture and compartmentalization. The capacity of this technology to reveal the molecular signatures of specific cell types and states under various pathological conditions makes it especially valuable for creating high-resolution cellular atlases that can facilitate mechanistic understanding, identification of therapeutic targets, and development of personalized treatment strategies for kidney diseases [4,5].

Kidney disease is a growing public health issue that affecting millions of people worldwide and showing an increasing incidence in both developed and developing countries. The renal disease continuum ranges from acute injury to irreversible end-stage organ failure (ESRD), and is characterized by progressive and systematic destruction of both structural and functional renal units [6,7]. Although modern therapeutic arsenals, including angiotensin receptor blockers (ARBs), novel SGLT-2 inhibitors (SGLT2i), and biological immunotherapies, have demonstrated partial clinical efficacy [8], a significant proportion of patients still progress to dialysis-dependent states. This therapeutic limitation primarily originates from an insufficient mechanistic understanding of cell-type-specific disease pathways. Conventional bulk-tissue analyses, although instrumental in identifying broad pathogenic mechanisms, cannot adequately resolve the cellular heterogeneity in the kidneys. Renal tissue, which consist of multiple specialized cell types with compartment-specific injury responses, require precise interrogation to develop targeted therapies. Furthermore, the dynamic interplay between the resident and infiltrating cell populations during disease evolution necessitates the development of advanced analytical modalities [9]. These factors are driving new cell-type-selective and personalized therapeutic development paradigms.

Over the last decade, the advent of scRNA-seq in nephrology has revolutionized our understanding of the pathogenesis kidney diseases. Although several reviews have addressed various aspects of this technical revolution [4,10–13], systematic analyses of its developmental path, collaborative networks, methodological evolutionand, and future possibilities are lacking. Bibliometric analysis, which was developed by Alan Pritchard in 1969, provides a strong quantitative basis for mapping scientific advances and detecting emerging research frontiers [14]. Such analyses can also provide insights into publication trends, top contributors, knowledge networks, and institutions, offering great value in many biomedical fields [15,16]. A comprehensive bibliometric analysis of the application of scRNA-seq in kidney disease research, including the dynamics of publications, institution-based collaborations, and conceptual development, would facilitate an understanding of the developmental architecture of the field. Such an analysis, especially using keyword co-occurrence networks, can also reveal foundational principles, recent trends, and novel developments. This study seeks to address this crucial gap by performing an extensive bibliometric analysis with the following objectives: (1) to outline the evolution and current state of scRNA-seq applications in kidney disease research; (2) to identify primary contributors and collaborative networks, map conceptual frameworks, and methodological innovations; and (3) to delineate future research trajectories. These insights will provide a strategic outline for future ventures in this fast-moving domain.

## Methods

### Data source and search strategy

**Figure 1.**
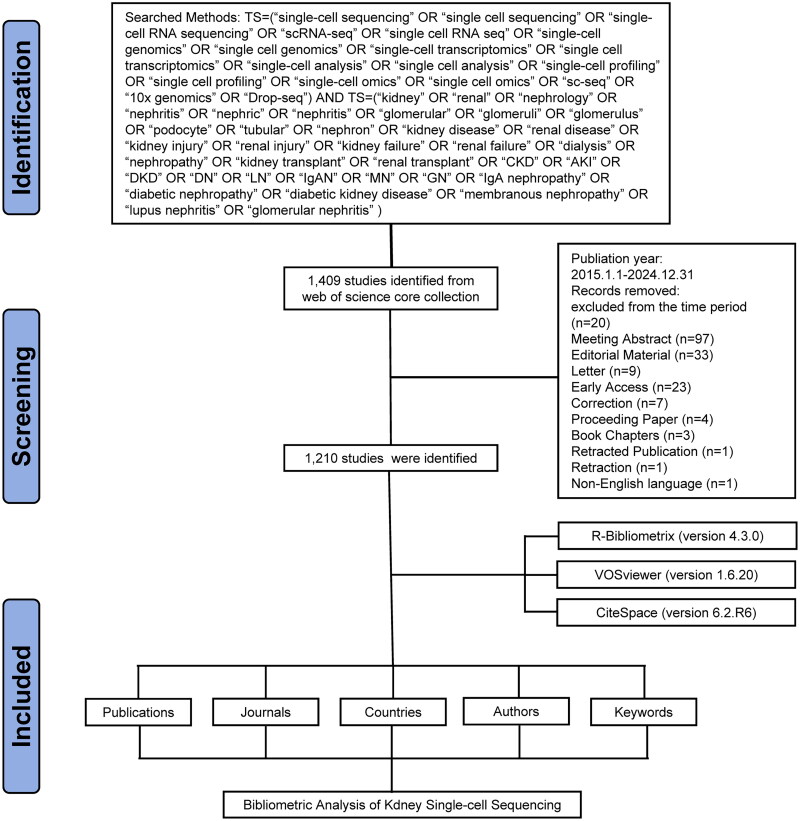
Literature screening process flowchart.

In the present study, we conducted a systematic bibliometric analysis to map the research landscape of scRNA-seq in the field of nephrology. We conducted an extensive search and analysis using the Web of Science Core Collection (WoSCC) database, covering the English-language literature from 2015 to 2024 (up to December 31, 2024). Bibliometric analysis is generally performed using the WoSCC databases because this databases cover several renowned and influential journals, favored by academic researchers [17]. Additionally, WoSCC offers comprehensive citation tracking, standardized data analysis, and consistent indexing practices, which are particularly beneficial for analyzing the rapidly evolving field of nephrology research. We employed the following search term combinations: [TS=(‘single-cell sequencing’ OR ‘single cell sequencing’ OR ‘single-cell RNA sequencing’ OR ‘scRNA-seq’ OR ‘single cell RNA seq’ OR ‘single-cell genomics’ OR ‘single cell genomics’ OR ‘single-cell transcriptomics’ OR ‘single cell transcriptomics’ OR ‘single-cell analysis’ OR ‘single cell analysis’ OR ‘single-cell profiling’ OR ‘single cell profiling’ OR ‘single-cell omics’ OR ‘single cell omics’ OR ‘sc-seq’ OR ‘10x genomics’ OR ‘Drop-seq’) AND TS=(‘kidney’ OR ‘renal’ OR ‘nephrology’ OR ‘nephritis’ OR ‘nephric’ OR ‘nephritis’ OR ‘glomerular’ OR ‘glomeruli’ OR ‘glomerulus’ OR ‘podocyte’ OR ‘tubular’ OR ‘nephron’ OR ‘kidney disease’ OR ‘renal disease’ OR ‘kidney injury’ OR ‘renal injury’ OR ‘kidney failure’ OR ‘renal failure’ OR ‘dialysis’ OR ‘nephropathy’ OR ‘kidney transplant’ OR ‘renal transplant’ OR ‘CKD’ OR ‘AKI’ OR ‘DKD’ OR ‘DN’ OR ‘LN’ OR ‘IgAN’ OR ‘MN’ OR ‘GN’ OR ‘IgA nephropathy’ OR ‘diabetic nephropathy’ OR ‘diabetic kidney disease’ OR ‘membranous nephropathy’ OR ‘lupus nephritis’ OR ‘glomerular nephritis’)] to capture the full spectrum of kidney conditions and scRNA-seq. We developed a systematic keyword-search strategy to comprehensively address the technological dimensions of scRNA-seq and renal pathology. This dual-focused approach encompassed three methodological considerations: (1) integration of standardized abbreviations (CKD, AKI, DKD) with conceptual terms (renal/kidney/nephrology) to reconcile terminological variations across disciplines; (2) inclusion of disease-specific descriptors (IgA nephropathy, diabetic nephropathy) to enhance diagnostic specificity; and (3) application of scRNA-seq-related terms to capture evolving technical applications. We selected the period from 2015 to 2024 for our bibliometric analysis, because our search revealed scarce literature prior to 2015. All literature related to scRNA-seq in nephrology was exported from WoSCC in TXT format as ‘Full Record and Cited References.’ These bibliographic records were subsequently imported into the Bibliometrix R package, VOSviewer, and CiteSpace for comprehensive bibliometric analysis, as illustrated in Figure 1. Additional methodological flowcharts and detailed search processes are provided in the Supplementary Materials.

## Data analysis

We employed a suite of bibliometric tools to analyze and visualize our literature data: the Bibliometrix R package (version 4.3.0), VOSviewer (version 1.6.20), and CiteSpace (version 6.2. R6). The Bibliometrix R package, which was accessed through its web interface Biblioshiny, was used to conduct frequency analyses of publications, authors, journals, institutions, and countries. For each category, we displayed the top 20 frequencies and generated a word cloud for the 50 most frequently used author keywords. Data visualization was enhanced using the ggplot2 R package (version 3.4.2). VOSviewer, developed at Leiden University, was used to generate co-occurrence networks illustrating the relationships among the authors, countries, institutions, and keywords. In these networks, node sizes represent occurrence or citation frequencies, whereas link thicknesses indicate collaboration intensities. We set minimum occurrence thresholds of 10 for authors, institutions, journals, and keywords; five for countries; and 100 for article citations. For a deeper structural and temporal analysis, we employed CiteSpace, a pioneering bibliometric software developed by Professor Chaomei Chen [18], to perform keyword cluster analysis and timeline visualization. The analysis was performed using Pathfinder for network pruning and merging with keyword K-means clustering conducted using a G-index value of *k* = 10.

## Results

### Number of publications and citation evolution

**Figure 2.**
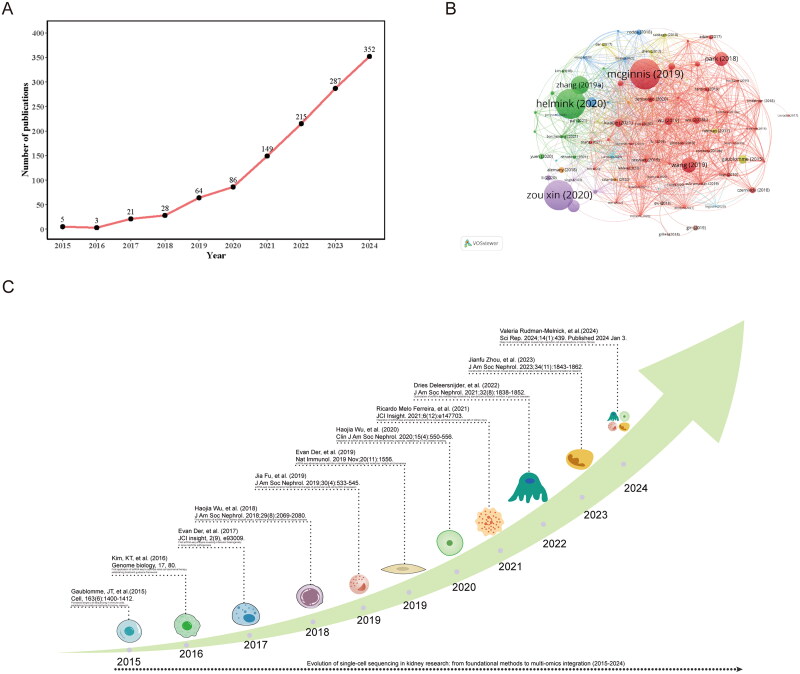
Publication analysis and timeline. **A** annual publication trends. **B** publication co-citation network. **C** milestone articles in kidney scRNA-seq research.

From the WoSCC, we identified 1,210 eligible publications, including1,095 original research articles and 115 review articles. A time-series line plot was constructed to illustrate the publication trends in the application of scRNA-seq in nephrology from 2015 to 2024 (Figure 2A). Analysis of the growth trajectories revealed two distinct phases: an initial steady growth phase (2015–2017), followed by a period of exponential expansion (2018–2024).

According to the co-citation network analysis performed VOSviewer (Figure 2B), the most highly cited paper in the WoSCC database was ‘DoubletFinder: Doublet Detection in Single-Cell RNA Sequencing Data Using Artificial Nearest Neighbors’ [19]by Christopher S. McGinnis, which was published in *Cell Systems* (impact factor [IF] = 9.0) in 2019 and had accumulated 1,566 citations. The second most cited paper was ‘Single-cell RNA-seq data analysis on the receptor ACE2 expression reveals the potential risk of different human organs vulnerable to 2019-nCoV infection’ [20]by Zou Xin, which was published in *Frontiers of Medicine* (IF = 3.9). Subsequently, we constructed a chronological timeline highlighting the key milestones of these influential papers [21–30] by evaluating their citation counts and relevance to scRNA-seq and kidney disease (Figure 2C).

### Core journal analysis

**Figure 3.**
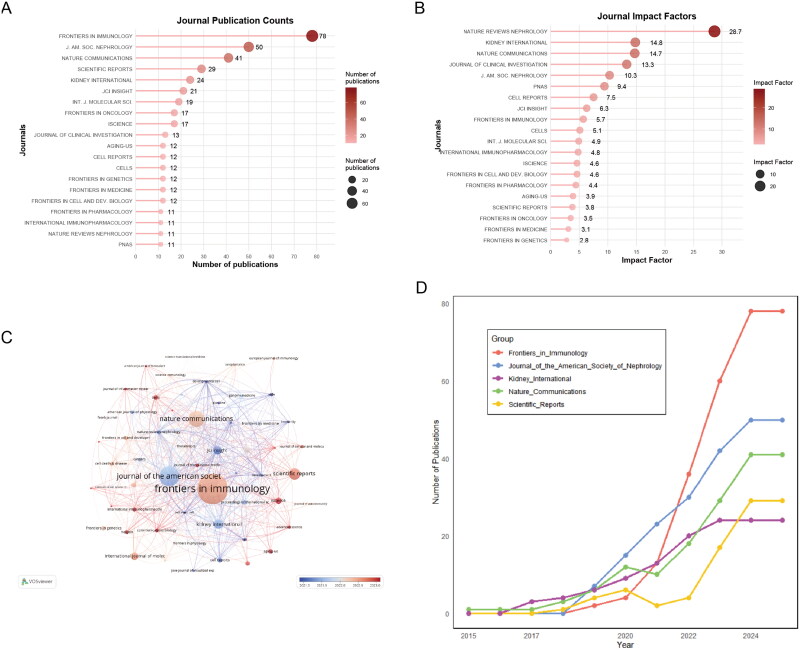
Journal impact assessment. **A** top 20 journals by publication volume. **B** Journal rankings by H-index. **C** Journal co-citation analysis. **D** publication evolution in leading journals.

Figure 3A illustrates the top 20 journals that published articles describing the use of scRNA-seq in kidney disease reaseach between 2015 and 2024. These journals collectively published 424 articles, accounting for 35.19% of all publications on this topic. Among these, *Frontiers in Immunology*, with an IF of 5.7, ranked Q1 in Journal Citation Reports and published 78 relevant papers, representing 6.47% of all published articles. The H-index, a metric used to evaluate both the quantity and impact of publications, showed that *Nature Reviews Nephrology* (H-index = 28.7) and *Kidney International* (H-index = 14.8) were the two most influential journals in this field, as shown in Figure 3B. Furthermore, the co-citation network of journals visualized using VOSviewer (Figure 3C) indicated that the three key journals with the highest total link strengths were *Journal of the American Society* of *Nephrology*, *Frontiers in Immunology*, and *Nature Communications*.

### Analysis of countries and institutions

**Figure 4.**
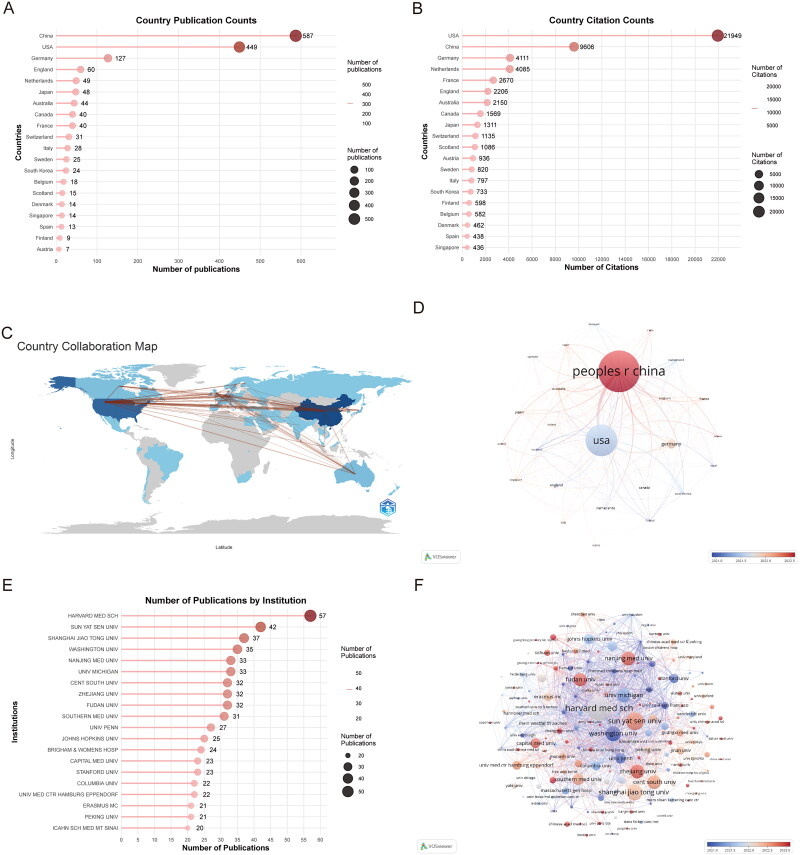
Contribution and collaboration among countries and institutions. **A** leading countries by publication count. **B** countries ranked by citation impact. **C** international research networks. **D** global collaboration map (>10 publications threshold). **E** leading research institutions. **F** institutional partnerships (>5 publications threshold).

Figure 4A and B illustrate the publication output and collaborative networks among the 58 countries engaged in scRNA-seq research on kidney disease. In terms of productivity, China led with 587 publications, followed by the United States (449 publications), Germany (126 publications), the United Kingdom (60 publications), and the Netherlands (49 publications). In terms of citation impact, the United States demonstrated remarkable performance in this research field with 21,949 citations, surpassing other countries such as China (9,606 citations), Germany (4,111 citations), and the Netherlands (4,085 citations). Using VOSviewer and Bibliometrix R, Figure 4C and D reveal the collaborative relationships among major countries, highlighting the extensive cooperation and exchange among China, the United States, and Germany. Among the 25 countries with at least five internationally collaborative publications, the United States showed the highest number of international collaborations (323), followed by Germany (173) and China (158).

These publications originated from 1,689 institutions worldwide. Figure 4E presents the top 20 most productive institutions in scRNA-seq research on kidney diseases; the list included nine institutions each from China and the United States. The remaining two institutions were the University Medical Center Hamburg-Eppendorf in Germany and Erasmus MC in the Netherlands. Harvard Medical School led in terms of publication output with 57 articles, followed by Sun Yat-sen University (42 articles), Shanghai Jiao Tong University (37 articles), and Washington University (35 articles). The collaboration network shown in Figure 4F highlights the most influential institutions, including Harvard Medical School (196 links), University of Michigan (98 links), and Johns Hopkins University (96 links).

### Author contributions

**Figure 5.**
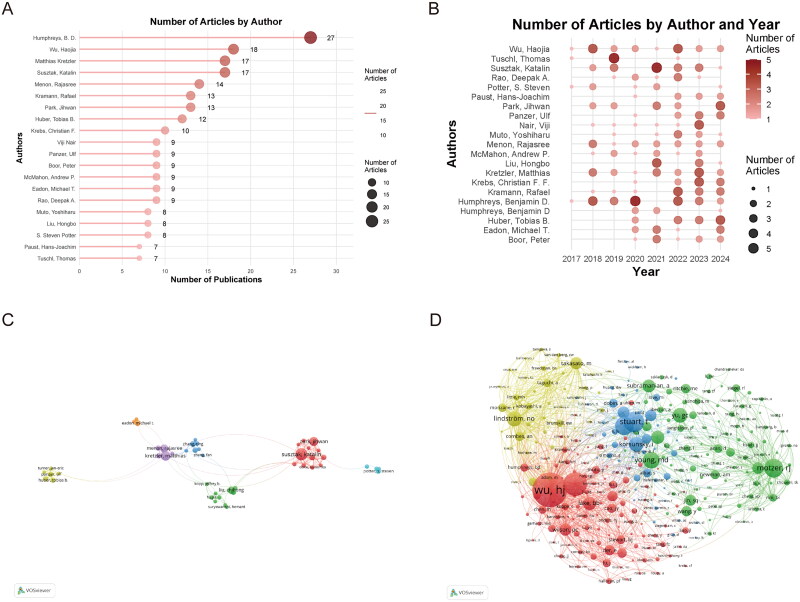
Authorship analysis. **A** the leading contributors. **B** the author productivity timeline. **C** research collaboration network. **D** author impact network.

The scRNA-seq literature on kidney disease has drawn contributions from 8,984 authors, averaging 7.44 authors per publication. The 20 most prolific authors collectively produced 233 articles, constituting 19.32% of the total publications in this field (Figure 5A). B. D. Humphreys of Washington University was the leading contributor, with 27 publications, followed by his colleague Haojia Wu (18 publications) and Matthias Kretzler (17 publications) from the University of Michigan. The publication trajectory of the top 20 authors, illustrated in Figure 5B, demonstrates their consistent scholarly output and sustained commitment to advancing the field. To elucidate key collaboration patterns, we generated a collaboration network using VOSviewer (Figure 5C). The network visualization, which focused on 114 authors with a minimum of five publications each, revealed robust collaborative relationships across research groups. Network analysis showed centrality measures below 0.1 for all authors, with Susztak Katalin exhibiting the highest collaboration intensity (link strength: 64), followed by Joshua D. Ooi and Yong Zhong (link strength: 54 for both). The co-citation network analysis encompassed 285 authors who received at least 20 citations (Figure 5D), and it identified Haojia Wu, Park, Jihwan, and Nils O. Lindstrom as the most influential authors based on their citation patterns.

### Keyword and hotspot analyses

**Figure 6.**
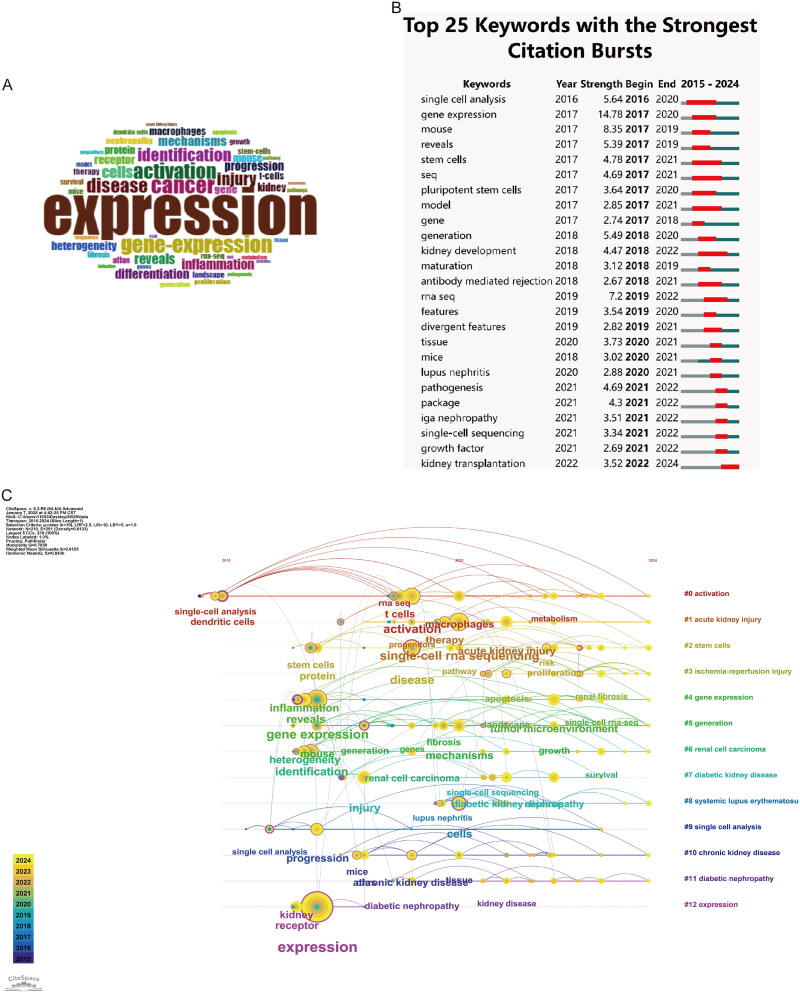
The research focus evolution. **A** the keyword frequency visualization. **B** the timeline of keyword clustering. **C** trend topics based on author’s keywords over time.

Keyword analysis of studies using scRNA-seqs in kidney disease research highlighted several key themes (Figure 6A). The predominance of ‘expression’ (*n* = 290) and ‘gene-expression’ (*n* = 105) reflects the central role of transcriptional profiling in understanding kidney cell heterogeneity and function. Disease-related terms were prominent, with ‘cancer’ (*n* = 107) and ‘disease’ (*n* = 91) emphasizing the technology’s applications in studying kidney pathologies. The frequent occurrence of ‘activation’ (*n* = 97) and ‘identification’ (*n* = 81) underscored the importance of characterizing cellular states and identifying distinct cell populations in kidney tissue, which is crucial for understanding disease progression and developing targeted therapies. The CiteSpace burst-detection analysis further identified emerging research trends, highlighting the increasing attention to ‘single-cell sequencing’, ‘growth factor’, and ‘kidney transplantation’, as demonstrated in Figure 6B. The temporal evolution of keyword clusters is visualized in Figure 6C. Initial research primarily focused on exploring basic molecular mechanisms, with emphasis on fundamental studies such as ‘gene expression’ and ‘identification’ Subsequently, the research focus gradually shifted toward in-depth investigation of specific diseases, particularly in clinically relevant areas such as ‘acute kidney injury’ and ‘diabetic nephropathy’, while simultaneously addressing key pathological mechanisms, including ‘inflammation’ and ‘oxidative stress pathways’. More recently, scNRA-seq technology applications have become increasingly sophisticated and mature, as evidenced by the tight integration of technologies such as ‘single-cell RNA sequencing’ and ‘single-cell analysis’ with specific disease studies. The research scope has further expanded to encompass broader disease areas, including ‘renal fibrosis’ and ‘chronic kidney disease’. Notably, studies have increasingly focused on cellular heterogeneity and the role of specific cell populations such as ‘macrophages’ and ‘T cells’, demonstrating a deepening understanding of cellular complexity in kidney diseases.

**Figure 7.**
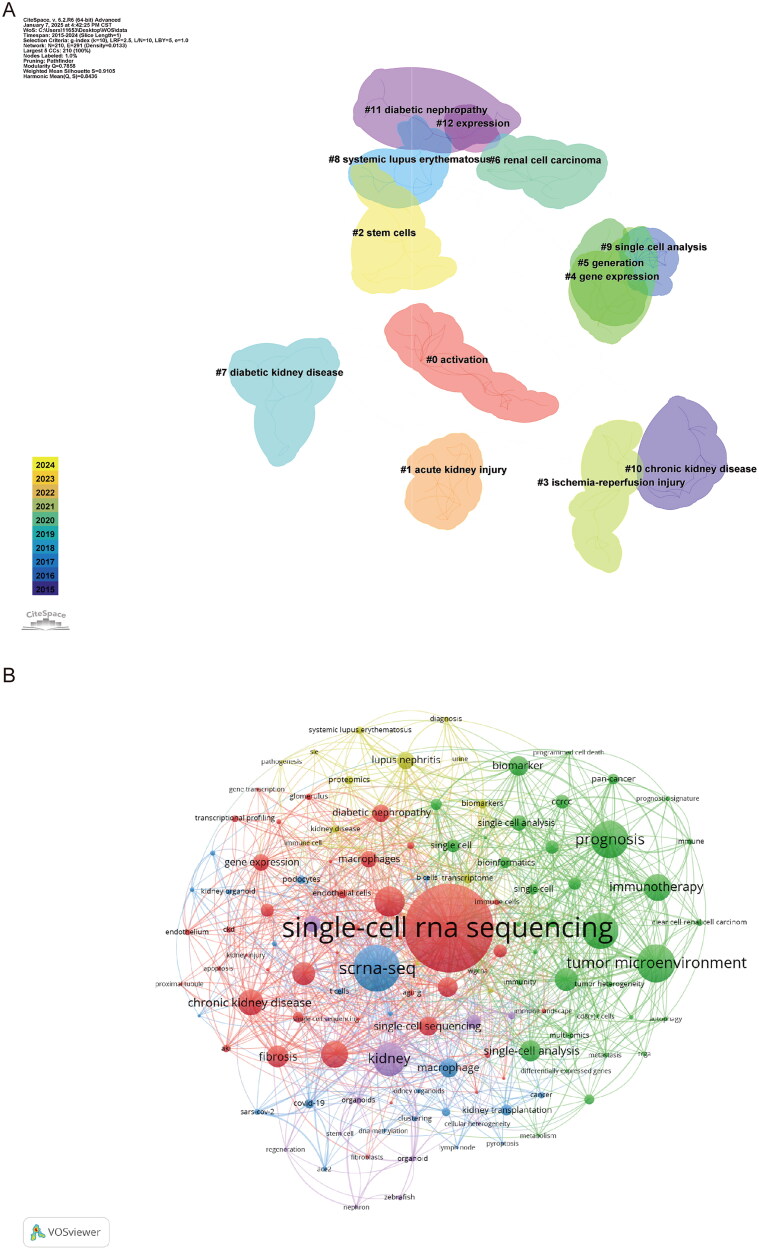
Keyword network analysis. **A** the keywords of citeSpace cluster analysis. **B** the keyword co-occurrence map.

We performed keyword analysis on 1,210 selected articles using VOSviewer and CiteSpace. To examine the relationships between keywords and to identify common themes, we conducted a co-occurrence analysis. Using CiteSpace, we divided these keyword clusters into 12 distinct categories: activation, acute kidney injury, stem cells, ischemia-reperfusion injury, gene expression, generation, renal cell carcinoma, diabetic kidney disease, systemic lupus erythematosus, single-cell analysis, chronic kidney disease, diabetic nephropathy, and expression (Figure 7A). Similarly, using VOSviewer, we constructed a co-occurrence network from the top 124 keywords that appeared at least five times (Figure 7B) and identified clusters represented by different colors, including red, green, blue, and yellow. Nodes of the same color within a cluster represent closely related co-occurrence relationships, with the node size and link width reflecting the strength of these associations. ‘Betweenness centrality’ was used to measure the importance of nodes in the network, with larger nodes indicating a higher total link strength and reflecting greater significance in the research field. In this network visualization, ‘single-cell RNA sequencing’ appeared to be the largest central node, indicating its pivotal role as a methodological approach. The clusters revealed distinct research themes: the red cluster encompassed chronic kidney disease, fibrosis, and gene expression; the green cluster focused on prognosis, immunotherapy, and the tumor microenvironment; the blue cluster included macrophages and scRNA-seq methodologies; and the yellow cluster was primarily related to lupus nephritis and systemic lupus erythematosus. The thickness of the connecting lines between nodes provides a clear visualization of the strength of the relationships between research themes.

## Discussion

With technological advancements, scRNA-seq technology has garnered increasing attention in the medical field, establishing itself as a significant focus of research. Furthermore, the application of scRNA-seq technology has brought cellular heterogeneity in kidney disease to the forefront of researcher interest [31,32]. This study represents the first attempt to employ bibliometric analysis to investigate publications describing the use of scRNA-seq in kidney disease research. We conducted a bibliometric visual analysis of 1,210 documents extracted from the WoSCC database using R software, VOSviewer, and CiteSpace. The results of this study provide a comprehensive understanding of the research trends in scRNA-seq analyses of kidney diseases from 2015 to 2024. Based on the publication output trends, articles related to scRNA-seq in kidney disease research have shown steady growth since 2018, with explosive growth occurring since 2020. This surge can be attributed to the publication of key articles and technological advancements that have driven interest in this field. Analysis of journal publications revealed considerable enthusiasm among international journals, particularly specialized journals such as *Frontiers in Immunology*, *Journal of the American Society of Nephrology*, and *Nature Communications*, for exploring scRNA-seq in kidney diseases. This indirectly reflects the strong interest of prestigious journals in this research area. Scientific leadership and international collaboration patterns in scRNA-seq kidney research have revealed distinct characteristics across nations.

This complex picture is consistent with what we expected; while China dominates in terms of the volume of research output, the United States sustains a noteworthy citation impact, which reflects more nuanced dynamics behind the research impact. The strong impact of citations from the United States can be attributed to several key factors. Our analysis revealed significant differences in international collaboration rates, creating broader citation networks for publications from the United States. Examination of innovation indices showed the publications from the United States more frequently introduced novel methodological approaches, while pioneering discoveries emerged from sustained investments in next-generation scRNA-seq technologies and analytical platforms [33]. Furthermore, established research institutions provided robust funding support and infrastructure [34], which was complemented by the nation’s comprehensive international collaboration network that effectively integrates diverse expertise and resources [35]. On the other hand, the increasing number of published studies from China reflects the rapid advancement of scRNA-seq technology in China and its substantial investments. The overall trends indicate very strong domestic research output and innovation in this area; however, we see indicators of international collaboration that can be accelerated to improve China’s global impact, especially in comparison with the networks in the USA and Germany. Research Brian tracks research impact metrics, which show that Germany and the Netherlands are at the top in terms of research impact. However, through substantial collaborations with US research teams and international groups, these countries have also assumed key leadership roles in the application of scRNA-seq in the field of kidney diseases.

The results of publication analysis indicated that B. D. Humphreys from Washington University was a highly prominent researcher in kidney disease research using scNRA-seq. His scientific leadership, combined with the pioneering leadership of Washington University in the development of scRNA-seq technology for application in nephrology, has placed this subfield on the trajectory it has followed since its implementation. Together with his long-time collaborator Haojia Wu, he laid the fundamental methodological frameworks that propelled rapid development in this field. The studies published by this author encompass important areas in nephrology with a broad focus on fundamental mechanisms clinical applications advances in technology, and therapeutic approaches, illustrating a global research mentality [28,36–39]. In this regard, a temporal analysis of author productivity also highlights a favorable trend: persistence from top-tier investigators indicates the transition from initial mechanistic dabbling to established research programs with a firm footing in understanding cellular heterogeneity in kidney diseases. Notably, Susztak Katalin’s position as the most interconnected researcher demonstrates the power of collaborative leadership in advancing the field, particularly in utilizing scRNA-seq methods to elucidate the molecular mechanisms of kidney diseases and translating research findings into clinical applications [29,40,41]. Similarly, the strong partnership between Joshua D. Ooi and Yong Zhong illustrates how cross-institutional collaboration can accelerate scientific progress through complementary expertise [42,43]. The co-citation patterns further illuminate the field’s intellectual structure, and researchers such as Haojia Wu, Park Jihwan, and Nils O. Lindstrom are key influencers whose their methodological innovations and research findings have become fundamental references, guiding subsequent investigations in kidney disease single-cell analysis and shaping the field’s theoretical framework [44–46].

We must also acknowledge the inherent limitations and shortcomings of bibliometric approaches. First, publication bias likely influenced our analysis, since positive findings are more frequently published than negative or inconclusive results, potentially skewing the apparent research landscape. Second, although comprehensive, the WoSCC database may underrepresent contributions from emerging economies and non-English publications. Third, although informative, citation metrics imperfectly measure the research impact and may be influenced by self-citation practices and research network effects. Furthermore, the lag between research completion and publication indicates that our analysis likely underestimated recent technological developments. Finally, bibliometric approaches primarily capture the explicit knowledge expressed in publications, potentially overlooking tacit knowledge and unpublished methodological innovations circulating within the research community.

### Technological advances in single-cell sequencing for kidney research

According to our bibliometric analysis, scRNA-seq platforms have evolved from isolated applications to achieving multidimensional integration in kidney research, demonstrating a clear trajectory from technical exploration to mature implementation. Each phase of this evolution is characterized by distinctive technological breakthroughs and innovative applications that have substantially accelerated our understanding of kidney diseases and their underlying mechanisms. Table 1 presents the development history of scRNA-seq technologies, which has been summarized in the following paragraphs [47–78].

**Table 1.** Development history of single-cell sequencing platform technology.

Year	Technology name	The advantages
2009	Tang method	The first realization of scRNA-seq
2011	STRT-seq	Preferentially at the 5′end of the mRNA
2012	Cel-seq	Linearly amplifying mRNA
2012	Smart-seq	Improved read coverage across transcripts
2012	Fluidigm C1	The first commercial platform
2014	Smart-seq2	The generation of full-length cDNA and sequencing libraries
2014	MARS-seq	Automated massively parallel scRNA-seq
2015	Cytoseq	A standard technology for high-throughput detection of protein markers on single cells
2015	Drop-seq	Quickly profiling thousands of individual cells
2015	inDrop-seq	Using microfluidic droplet technology to achieve high-throughput sequencing
2015	RNA-printing	Capture RNA through solid-phase and print RNA on glass
2016	Cel-seq2	First single-cell, on-chip barcoding method
2016	LCM-seq	Fixed cells are subjected to direct lysis without RNA extraction
2016	Spatial-seq	Visualization and quantitative analysis of gene expression
2017	sciRNA-seq	Combinatorial indexing, analyze the tens of thousands of single cells
2017	Seq-Well	Portable, low-cost platform and massively
2017	10× Chromium	High-throughput, rapid cell encapsulation and high cell capture efficiency
2017	MATQ-seq	A highly sensitive and quantitative method
2018	scNMT-seq	Parallel profiling of chromatin accessibility, DNA methylation and transcription in single cells, providing a comprehensive multi-omics view
2018	Microwell-Seq	Convenient, low-cost, and robust platform for high-throughput
2018	SpliT-seq	Efficient sample multiplexing and requires no customized equipment
2019	BD Rhapsody	High-throughput, high purity cell capture, low multiplet rate and multitier barcoding scheme
2019	scChIC-Seq	Profiling chromatin states at single-cell resolution
2020	Direct-seq	Simultaneously profiling CRISPR perturbations and their transcriptional readouts in a streamlined workflow without reverse transcription primers.
2020	scPINN-seq	Integrating single-cell RNA sequencing data with protein-protein interaction
2020	SHARE-seq	Simultaneous profiling of chromatin accessibility and gene expression
2020	Smart-seq3	Coverage with a 5′ unique molecular identifier for RNA counting to reconstruct thousands of RNA molecules
2021	Chromium iX	Economical and flexible for single-cell analysis from low to high throughput
2021	Chromium X	Capable of processing millions of cells at once with stability and cost-effectiveness
2022	Stereo-seq	Integrates high-resolution spatial transcriptomics with a large field of view for mapping cellular heterogeneity
2022	ISSAAC-seq	A highly sensitive and flexible single-cell multi-omics method
2022	CosMx SMI	enables high-plex imaging of RNA and proteins at subcellular resolution
2023	Xenium	High sensitivity and specificity, with high resolution and spatial resolution capabilities
2023	PIP-seq	Supports various emulsification formats, enabling rapid processing of thousands of samples or millions of cells
2024	PopV-seq	Integrating multiple algorithms to accommodate various cell labeling needs, data processing efficiency is improved

The pioneering phase of scRNA-seq, (2009–2015): In 2009, Tang et al. introduced the first methodology, namely, Tang’s methodology, for analyzing single-cell transcriptomes, which was followed by the STRT, CEL-seq, and Smart-seq platforms. Each of these early platforms improved single-cell transcriptomes analysis. Smart-seq and its optimized version, Smart-seq2, have become widely used platforms, offering near-full transcript coverage and enabling in-depth investigation of gene isoforms and splice variants [51,79]. However, these early methods had notable limitations, particularly in terms of throughput capacity and cost-effectiveness. The high per-cell processing costs and reduced cell capture capacity often limited research to small, focused cell populations. These limitations were partly alleviated by the introduction of multiplexing methods, including MARS-seq and Drop-seq in 2014–2015, and droplet-based approaches. Although limited from a technical perspective, these early technologies set the stage for cellular heterogeneity analysis and paved the way for many high-throughput methodological breakthroughs that soon followed.

scRNA-seq technology has progressed through several stages, each characterized by a breakthrough in the technology and/or resolution of previously existing technical barriers. A major turning point occurred between 2016 and 2017 with the advent of spatial and high-throughput techniques, most notably the 10x Chromium platform. This advancement marked a pivotal transition from expensive low-throughput methods to scalable practices [80,81]. After 2017, technological developments accelerated, and high-throughput platforms (MATQ-seq, scNMT-seq, and Microwell-Seq) were designed to specifically overcome certain technical hurdles.

Most recently (2020–2024), the emphasis has turned to more multi-modal integration and higher spatial resolution, as seen in platforms such as SHARE-seq, Stereo-seq, and, more recently, Xenium and PIP-seq. From these early platforms, the field has rapidly matured, and throughput and cost barriers have been surpassed by current technologies offering unprecedented scale—both in terms of the number of cells interrogated and the resolution and multimodality facilitated—not only reshaping our understanding of cellular heterogeneity, but also enabling its dissection at single-cell resolution [62,82]. In addition to developments in sequencing platforms, improvements in sample-preparation protocols are important for broadening the application of these technologies in kidney research. Nuclei isolation approaches that minimize the degradation of RNA and the release of contaminating components, such as proteins and mRNA have become available, enabling high-efficiency, high-quality, single-nucleus RNA extraction from mixed tissues. Rousselle et al. developed a single-nucleus RNA isolation protocol tailored for clinical specimens. With a focus on frozen and RNA-preserved human kidney biopsy samples, their method of analysis significantly reduced cell aggregation and ultracentrifugation steps, allowing for sample processing in 90 min, while simultaneously preserving structural integrity and improving the quality and purity of RNA [83].

This method worked very well in identifying tubular cell types and was able to distinguish 16 different tubular cell types. Chih-Yang et al. took the field one step further by establishing a new nuclear-isolation protocol specifically for frozen mouse kidney tissue. Their method utilized joint mechanical and enzymatic dissociation, followed by flow cytometry-based sorting of isolated nuclei to extract high-quality nuclear RNA. The protocol was generally robust and effective in other complex tissues such as the placenta and pancreas, in addition to mouse kidney tissues [84]. In their landmark study, Haojia Wu et al. used the Drop-seq platform to recover ten separate cell clusters from adult kidney biopsies, highlighting previously unexplored cell types such as activated primary proximal tubular cells. Their work demonstrated that single-nucleus RNA sequencing can reduce dissociation bias while remaining compatible with frozen samples and avoiding the transcriptional stress responses often associated with enzymatic dissociation [85]. These methodological objectives have considerably enabled the analysis of fibrotic kidney tissue, especially in chronic kidney disease, where cell isolation is difficult. Nuclear sequencing approaches have expanded the scope of cell-type identification while maintaining high data quality, thereby deepening our understanding of renal cellular heterogeneity. Nevertheless, technical challenges persist, particularly in isolating specific cell populations from highly fibrotic regions and minimizing background RNA contamination. Addressing these limitations is crucial for further advances in single-cell analyses in kidney research [86].

### Applications of scRNA-seq in nephrology

**Figure 8.**
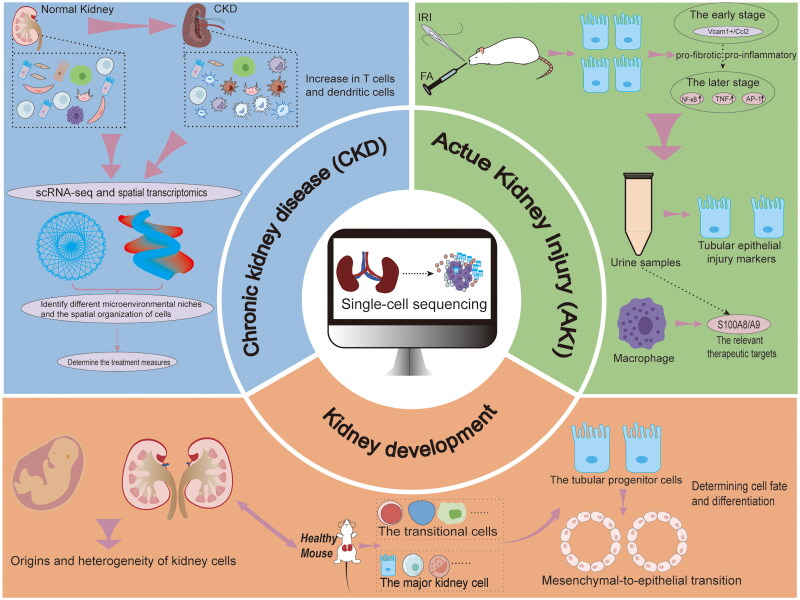
Applications of scRNA-seq in nephrology.

scRNA-seq has revolutionized our understanding of renal biology through its unprecedented resolution in studies of cellular heterogeneity and disease mechanisms. This technological approach has provided novel insights across the spectrum of nephrology, from developmental processes to pathological states, transforming the conceptual framework of kidney development, acute kidney injury (AKI) and CKD [87], as shown in Figure 8.scRNA-seq has revolutionized our understanding of kidney development by unveiling the complex cellular hierarchies and developmental trajectories during organogenesis. By tracking tubular progenitor cells through the mesenchymal-to-epithelial transition (MET), researchers have identified key transcription factors and signaling pathways crucial for cell fate determination and differentiation. For instance, pioneering scRNA-seq studies in healthy mouse kidneys established the first comprehensive single-cell transcriptomic atlas, revealing 21 distinct kidney cell types and identifying the developmental trajectories from transitional cells to various cell types in the kidney [86]. These findings were complemented by scRNA-seq analyses of fetal kidney tissue, which further illuminated the cellular origins and heterogeneity during renal development [88]. Notably, these studies identified a novel transitional cell population during MET, providing crucial insights into the stepwise progression of kidney development. Single-cell transcriptomics analyses have provided insights into cellular heterogeneity and the molecular mechanisms involved in AKI and repair. In ischemia-reperfusion injury (IRI) models, scRNA-seq analysis revealed distinct pro-inflammatory and pro-fibrotic proximal tubular cell populations characterized by Vcam1+/Ccl2+ expression with enhanced nuclear factor (NF)-κB and tumor necrosis factor (TNF) signaling activation [89]. The use of scRNA-seq in unilateral IRI (UIRI) models has further elucidated the pathogenic mechanisms of ischemic AKI, demonstrating that oxidative stress critically impairs proximal tubular cell repair by disrupting of cellular structure and function [90]. Although previous studies have mainly focused on ischemic injury, researchers have deepened our understanding of AKI in animal models through scRNA-seq analysis of folic acid-induced AKI. Through the construction of a comprehensive single-cell atlas containing 20 cell types with over 80,000 high-quality cells, researchers have identified seven different functional proximal tubular cell (PTC) subtypes, which revealed that, in comparison with IRI and cisplatin-induced AKI, folic acid-induced injury is more similar to unilateral ureteral obstruction, likely due to crystal-induced intrarenal obstruction [91]. More importantly, translating these experimental insights into clinical applications, single-cell analysis of urinary samples from AKI patients has further validated these findings, identifying diverse tubular epithelial cell populations exhibiting elevated levels of injury markers and pro-inflammatory cytokines, and thereby providing a noninvasive window into early kidney injury states [92]. Additionally, scRNA-seq identified a therapeutically relevant macrophage subpopulation (s14a8/a9hi) that constituted 47.69% of renal mononuclear cells with potent pro-inflammatory effects; targeting this subpopulation was shown to reduce mortality, AKI, and long-term renal fibrosis in experimental models [93]. While single-cell analysis of AKI has primarily revealed dynamic changes in tubular cell states and injury responses, its application in CKD has revealed more complex chronic alterations in the cellular landscape of the kidney. Using scRNA-seq analysis, researchers identified nine major renal cell populations, revealing a notable decrease in CD16+ natural killer (NK) cells and an increase in CD4+ naïve helper T cells and CCR7+ dendritic cells in patients with kidney disease [94]. Unlike the acute inflammatory response observed in AKI, these alterations in immune cell composition reflect persistent dysregulation of immune homeostasis, establishing a chronic pro-inflammatory environment that perpetuates disease progression [95]. The integration of scRNA-seq with spatial transcriptomics has revealed novel aspects of CKD pathogenesis that traditional scRNA-seq alone cannot reveal. As described by the authors themselves, this combined strategy allowed mapping of the spatial distribution of immune cells within the kidney and identified distinct microenvironmental niches that orchestrate local immune responses and tissue repair events *in situ*. Combined analyses revealed that specific stromal cell subtypes are the principal regulators of renal fibrosis, a hallmark of CKD, as opposed to AKI. These specialized stromal populations express unique transcriptional programs that drive progressive fibrosis through complex intercellular signaling networks, setting the stage for potential therapeutic targets to antagonize CKD progression. The increasing acknowledgment of the cellular and spatial complexity involved in CKD highlights the need to develop therapeutic strategies that target not only specific cell populations but also their complex interactions within the renal microenvironment [30]. Thus, as we continue to decode the cellular and molecular landscapes of both AKI and CKD using scRNA-seq, from initial injury responses to progressive structural alterations, the potential for precision medicine in nephrology has become increasingly tangible [30], offering hope for pathophysiology-based interventions and novel therapeutic strategies targeting disease-specific mechanisms.

The translation of scRNA-seq findings into clinical applications requires rigorous validation using multiple complementary approaches. Functional validation studies have substantiated the key pathogenic mechanisms initially identified through scRNA-seq, as exemplified by the validation of a specific pro-inflammatory macrophage subpopulation (s100A8/A9) through targeted depletion experiments, which confirmed its critical role in kidney injury pathogenesis and demonstrated significant therapeutic potential through improved survival metrics in experimental models [93]. Spatial validation methodologies, including immunohistochemistry, multiplexed *in situ* hybridization, and spatial transcriptomics, have provided crucial confirmation of cellular localization patterns within the complex kidney architecture, as evidenced by Melo Ferreira et al. [27], who verified the distinctive spatial distribution of inflammatory cells during injury, with neutrophils predominantly infiltrating the renal medulla and macrophages dominating the outer cortical regions. Translational validation studies have assessed the clinical utility of scRNA-seq biomarkers. A notably study in this regard was conducted by Klocke et al. [92], whose urinary scRNA-seq approach validated that transcriptional signatures initially characterized in experimental systems could be reliably detected in clinical specimens, establishing potential noninvasive monitoring methodologies. Furthermore, cross-platform validation employing orthogonal single-cell technologies has enhanced the robustness of scRNA-seq findings, with plate-based methodologies confirming droplet-derived data, and protein-level analyses *via* mass cytometry and single-cell proteomics corroborating the transcriptomic discoveries. Notwithstanding these major significant advances, comprehensive validation of the extensive array of scRNA-seq discoveries remains challenging owing to methodological limitations and resource constraints, underscoring the importance of standardized validation frameworks and integrated multi-omics strategies to maximize the translational impact.

### Spatiotemporal dynamics and disease evolution in kidney diseases

Spatiotemporal dynamics and disease progression represent a complex yet crucial area of research on kidney disease [4]. In this regard, the integration of scRNA-seq and spatial transcriptomics can not only compensate for the limitations of scRNA-seq in obtaining spatial information, but also, through the integration of multidimensional data and deeper analysis of cell-cell interactions, reveal temporal cascade events in kidney disease progression and spatial heterogeneity in cellular states and gene expression patterns. This integrated approach has significantly enhanced both the depth and breadth of research, providing a more comprehensive and precise tool for biomedical research.scRNA-seq technology can reveal cellular lineage trajectories at the individual cell level, enabling researchers to understand disease progression [12]. When integrated with spatiotemporal transcriptomics, this approach provides a more comprehensive identification of cell types across different spatial regions and temporal dimensions, while also detecting cell subpopulations that emerge only in specific spatial contexts [96]. In the trajectory analysis of disease progression, the integration with spatiotemporal transcriptomics can enable tracking of gene expression changes across different developmental stages or disease states, providing temporal information that helps researchers to understand disease-progression mechanisms more accurately, and records both the spatial distribution and temporal dynamics of cells [97]. Thus, this integration provides deeper insights into the spatial-distribution patterns and interactions of renal cells within tissues, yielding a more comprehensive understanding of cellular behavior and mechanisms during disease progression. For instance, integrated single-cell and spatial transcriptomic analyses of AKI have identified endothelial and stromal cells as early responders, followed by the upregulation of immune-related pathways in tubular epithelial cells [98]. Through optimized integration of spatial transcriptomics and scRNA-seq data, researchers successfully mapped 30 major cell types in human kidney tissue and revealed sequential responses in mouse AKI models, demonstrating the progression from initial rapid transcriptional changes in proximal tubular cells to subsequent immune cell recruitment and repair-mechanism activation, and immune cells showed distinct spatial-distribution patterns across different kidney regions, with specific chemokines (such as Atf3 and Mdk) expressed in epithelial cells facilitating immune cell infiltration and interaction, providing crucial molecular insights into the cellular responses following kidney injury [27]. Furthermore, scRNA-seq has been used to mapp the gradual transition of tubular epithelial cells from healthy to injured states in CKD patients. By identifying the key molecular switches controlling this transition, researchers particularly emphasized the importance of ‘checkpoint’ states in cellular repair and maladaptation, indicating that therapeutic interventions might be most effective within specific time windows [40]. In their study on diabetic nephropathy, Wilson et al. utilized single-nucleus RNA sequencing of 23,980 nuclei to reveal distinctive transcriptional trajectories in human kidney cortical tissue. Their analysis identified critical transition points characterized by coordinated changes in ion transport pathways and angiogenic signatures, which represented potential therapeutic windows for interventions before the onset of irreversible kidney damage [99]. Intercellular communication is a fundamental process in multicellular organisms that plays crucial roles in tissue development, homeostasis maintenance, and disease progression [100]. Although scRNA-seq technology can reveal differences in gene expression between cells, it cannot directly decode intercellular communication mechanisms [101]. Integration with spatiotemporal transcriptomics, by recording both cellular spatial localization and gene expression information, can enable clearer visualization of cell – cell interactions and communication networks [102]. Tools such as NicheNet utilize spatiotemporal transcriptomic data to decode intercellular communication relationships, thereby facilitating the construction of more precise cellular communication network models [101]. In kidney disease research, investigators have integrated scRNA-seq and spatial transcriptomics to identify distinct communication patterns between immune cells and epithelial cells during AKI, which revealed that neutrophils specifically infiltrate the renal medulla, while infiltrating macrophages dominate the outer cortex. The key chemokines Atf3 and Mdk were identified as critical factors that propagate and coordinate inflammatory responses within kidney tissue [27]. This integrated approach revealed how cells communicate through signaling molecules and enhanced the existing understanding of the influence of these communications on cellular functions and disease progression while identifying potential therapeutic targets [102]. By integrating single-cell sequencing and spatial transcriptomics technologies, a more comprehensive investigation into both the inter- and intra-tissue heterogeneity of diseases can be performed, enabling an in-depth analysis of gene expression patterns and functional variations across and within different tissue regions [27]. For instance, in lupus nephritis (LN) research, scRNA-seq technology has revealed the distribution and functional status of immune cells in kidneys. Spatial transcriptomics can further reveal that the distribution of different immune cell populations in different regions of the kidney varies significantly with disease progression and treatment response [103,104]. Therefore, combining scRNA-seq with spatial transcriptomics to achieve a comprehensive analysis of the molecular heterogeneity of kidney diseases can not only reveal the molecular characteristics of individual cells but also reveal the dynamic distribution and interactions of these characteristics in tissues through spatial localization, providing more precise targets for targeted therapy and thereby improving treatment efficacy and reducing side effects [105–107].

### Future perspectives: Challenges, opportunities, and interdisciplinary integration in single-cell nephrology

Despite the substantial significant advances in scRNA-seq technologies for kidney research, several challenges remain to be addressed, and new opportunities have emerged through interdisciplinary integration. The primary technical limitations of scRNA-seq in kidney research stem from the complex spatial architecture of the kidney, which includes diverse cell types and subtypes. While tissue dissociation into single-cell suspensions enables high-resolution transcriptional profiling, it inevitably disrupts the spatial relationships between cells, limiting our understanding of cell – cell interactions in their native context [108]. Recent advances have partially addressed this challenge by integrating spatial transcriptomics data using platforms such as Stellaris, which combines scRNA-seq data with spatial information from public databases to achieve more precise cellular localization and functional analysis. Single-nucleus RNA sequencing has been developed as a complementary method to eliminate the requirement for tissue dissociation, thereby maintaining spatial relationships in specific applications [85,109,110]. However, RNA degradation frequently occurs during nucleic acid extraction from frozen or fixed tissues [111]. Therefore, advances in cryopreservation technologies, such as programmable cooling devices and high-throughput vitrification methods, are being actively explored as alternatives to traditional slow-freezing approaches to reduce cellular damage and improve recovery rates [112,113]. Additionally, the standardization of analysis pipelines across different platforms and laboratories requires further optimization to ensure the reliability and comparability of research findings [114,115]. Data analysis and integration also present significant challenges in this field [116]. As single-cell datasets grow in complexity and volume, particularly when integrated with spatial transcriptomics and other omics data, they demand increasingly sophisticated computational approaches [117]. Although artificial intelligence and machine learning have demonstrated remarkable potential in scRNA-seq data analysis [118], the standardization of these methods and the validation of their predictions remain major challenges in current research, as evidenced by the significant data inconsistencies arising from variations in experimental conditions, sample sources, and sequencing technologies; thus, novel algorithms and tools are required for processing data from diverse sources [119,120]. Furthermore, with advancements in scRNA-seq technologies, multi-modal data integration has attracted growing interest; however, substantial differences in feature distributions and scales between different modalities, such as the simultaneous analysis of gene expression (scRNA-seq) and chromatin accessibility (scATAC-seq), continue to pose challenges for effective data integration [121]. The integration of scRNA-seq with other emerging technologies, particularly in multi-omics approaches combining transcriptomics, proteomics, metabolomics, and epigenomics at the single-cell level, can provide unprecedented insights into the cellular regulation of kidney diseases. Previous studies have demonstrated that advances in single-cell multi-omics technologies can enable the simultaneous analysis of genomic, transcriptomic, epigenomic, and proteomic information from the same cell, thereby revealing the complexity of cellular heterogeneity and function [122]. These technological advances have not only facilitated the identification of novel therapeutic targets and biomarkers but have also enhanced our understanding of cellular states and functions in kidney diseases [123]. Furthermore, the combination of single-cell transcriptomics and proteomics has been employed to investigate epigenetic regulation during kidney development [124], and the integration of diverse single-cell data types has improved our understanding of intercellular signaling and interactions [125]. However, this technological convergence faces several challenges, including accurate integration and interpretation of complex information from different data types and the existing limitations in integrating epigenomic information [126,127]. Therefore, to fully harness the potential of these technologies, advancements in computational methods and experimental strategies are essential for integrating and analyzing these complex multi-omics datasets [126]. The integration of single-cell technology with other disciplines, such as the combination of single-cell analysis with organoid-on-chip technology and 3D bioprinting, will enable the creation of more sophisticated models for drug screening and disease modeling [128–130], allowing researchers to precisely recreate cellular distribution and interactions in three-dimensional space and thereby better simulating disease states and facilitating drug screening for the development of more effective therapeutic strategies [131]. The clinical translation of scRNA-seq technology presents both challenges and opportunities. Although numerous potential therapeutic targets and biomarkers identified using scRNA-seq technologies require validation in larger patient cohorts, the development of cost-effective diagnostic tools remains a critical issue [132]. Additionally, sample collection and preservation, the limited sensitivity of analytical methods, and the complexity of translating single-cell data into clinically measurable parameters necessitate the establishment of standardized sample processing protocols and novel computational tools to integrate and analyze the data for simplified clinical interpretation [133]. Despite these translational challenges, the potential of the scRNA-seq technology in precision medicine cannot be overlooked. scRNA-seq has already demonstrated utility in monitoring disease progression, evaluating treatment responses, and predicting patient outcomes [134]. Furthermore, when combined with artificial intelligence algorithms, scRNA-seq technology shows promise in enhancing the efficiency of disease diagnosis and treatment efficiency [135].

Several emerging trends hold promise in advancing this field of research. The development of *in situ* sequencing technologies may overcome the current limitations of spatial resolution, and improvements in sample-preservation and processing methods will enable more accurate analysis of clinical specimens. Integration with artificial intelligence is expected to enhance the ability to predict disease trajectories and treatment responses, potentially revolutionizing personalized medicine approaches in nephrology [136].

## Supplementary Material

Figure1-8.zip
