# Supplementary figures and images for: Kidney diseases and single-cell sequencing research: a bibliometric analysis from 2015 to 2024
Source: Ren Fail. 2025 Jun 23;47(1):2521457. doi: 10.1080/0886022X.2025.2521457 (PMC12893499; doi:10.1080/0886022X.2025.2521457)

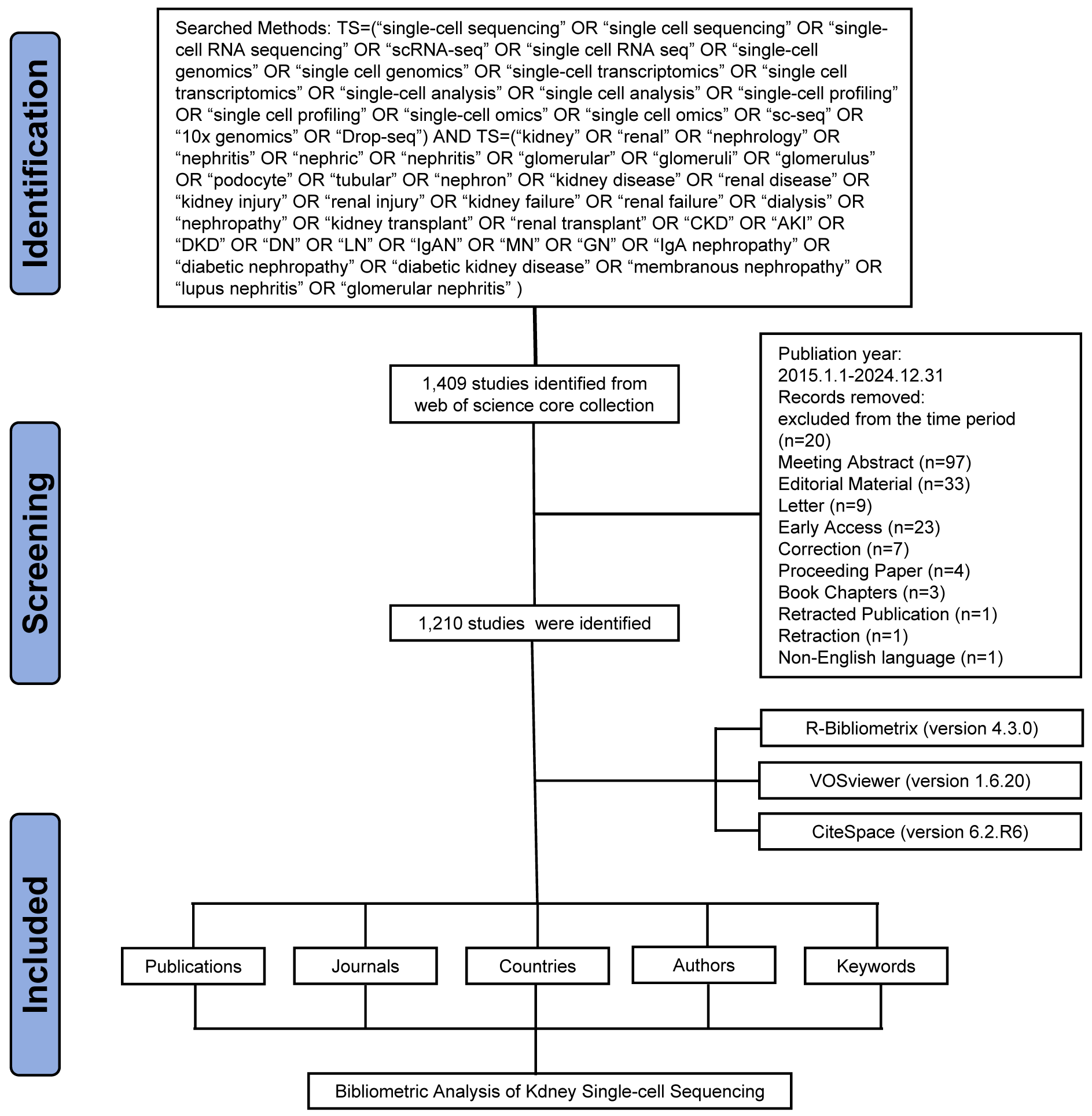

Supplement: Figure1-8.zip [file IRNF_A_2521457_SM2502.zip › Figure1-8/Figure1.tif]

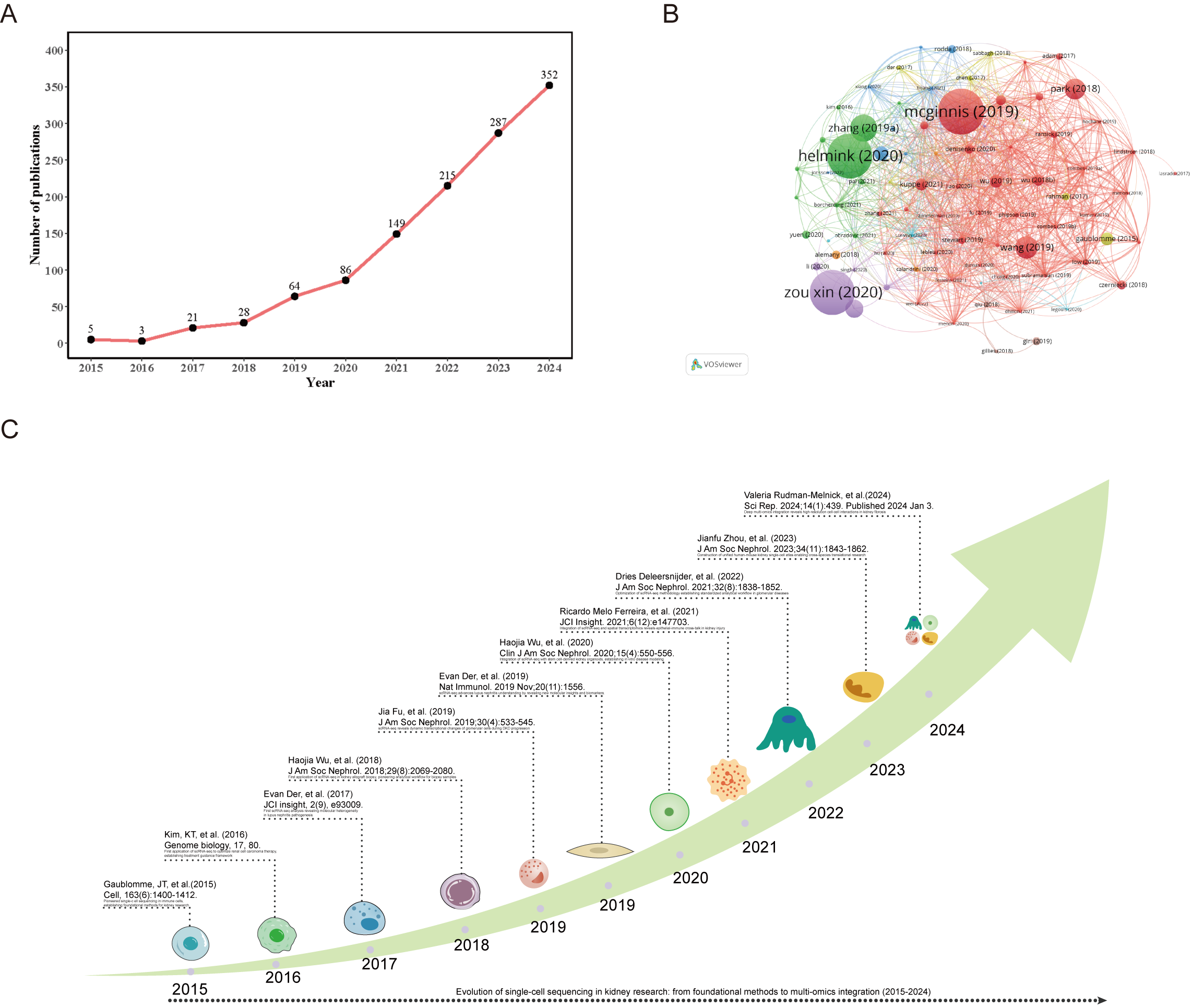

Supplement: Figure1-8.zip [file IRNF_A_2521457_SM2502.zip › Figure1-8/Figure2.tif]

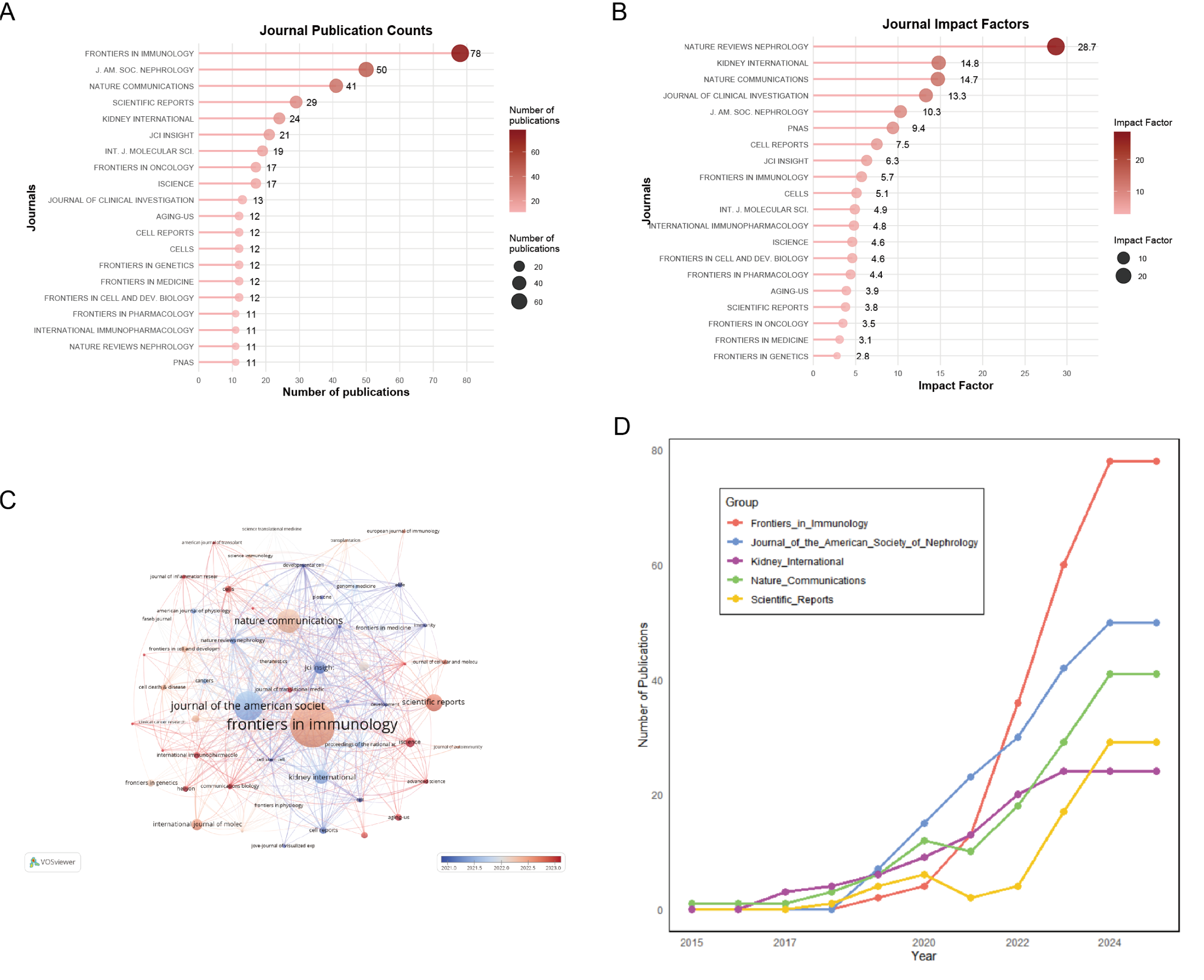

Supplement: Figure1-8.zip [file IRNF_A_2521457_SM2502.zip › Figure1-8/Figure3.tif]

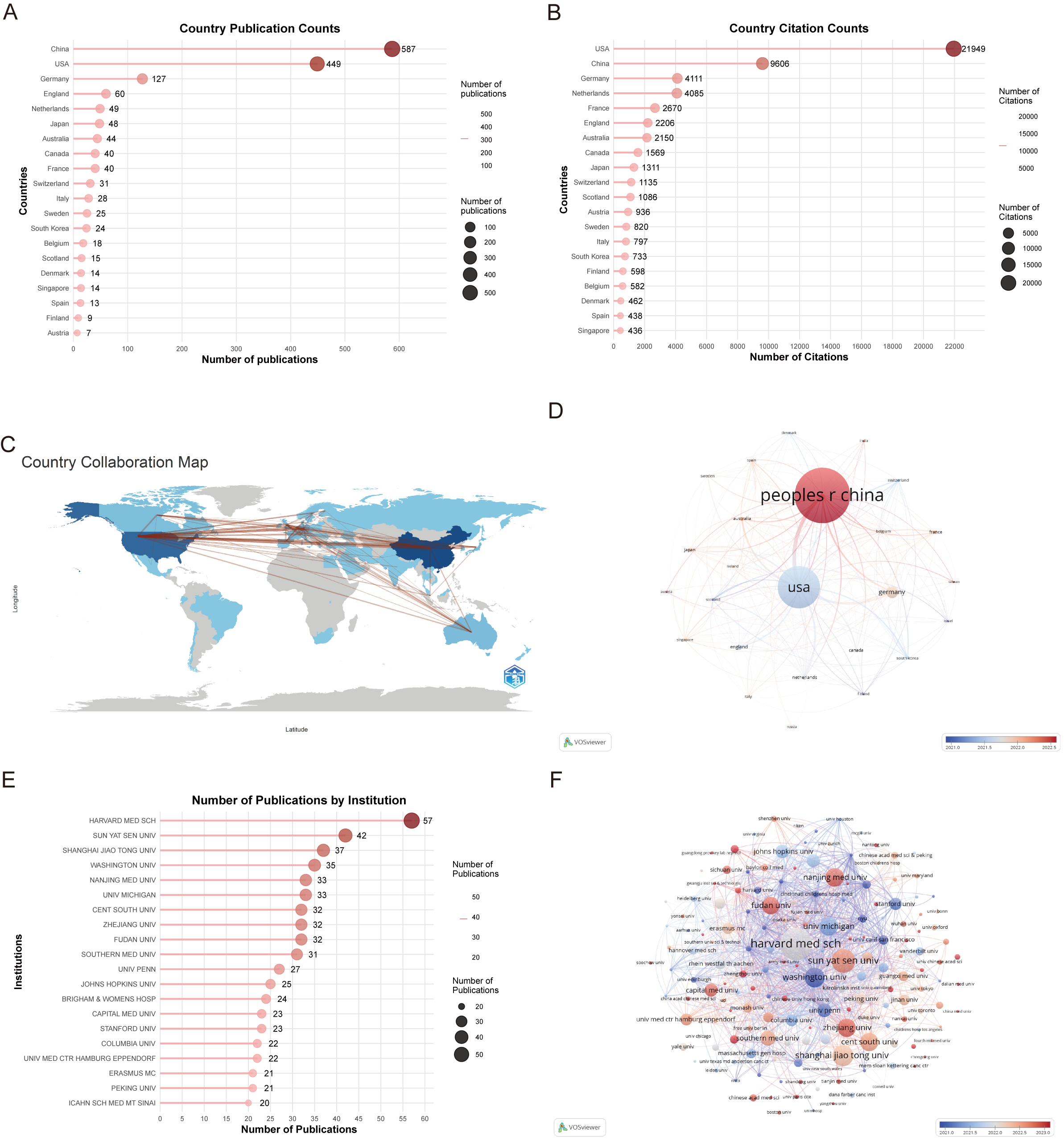

Supplement: Figure1-8.zip [file IRNF_A_2521457_SM2502.zip › Figure1-8/Figure4.tif]

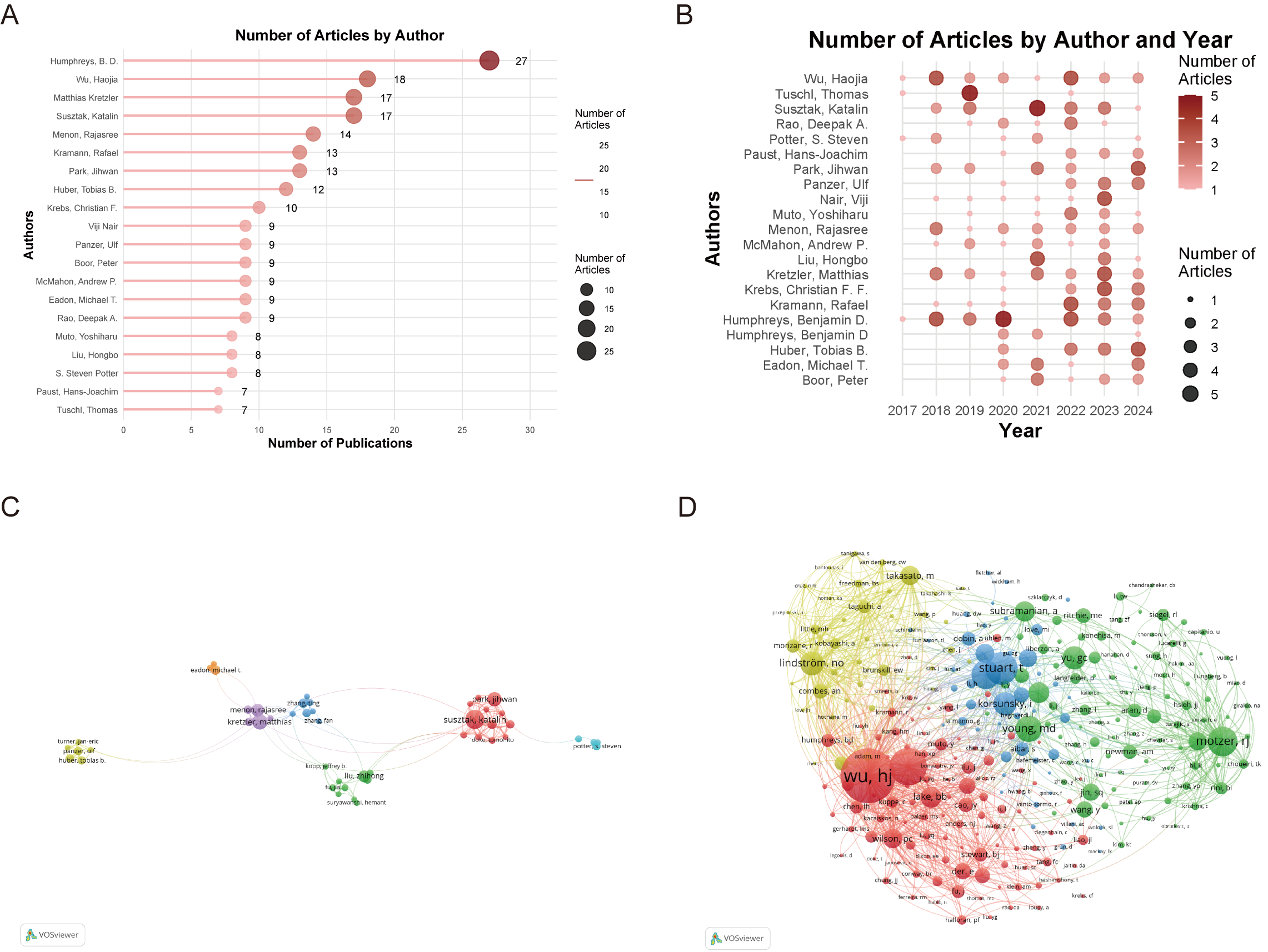

Supplement: Figure1-8.zip [file IRNF_A_2521457_SM2502.zip › Figure1-8/Figure5.tif]

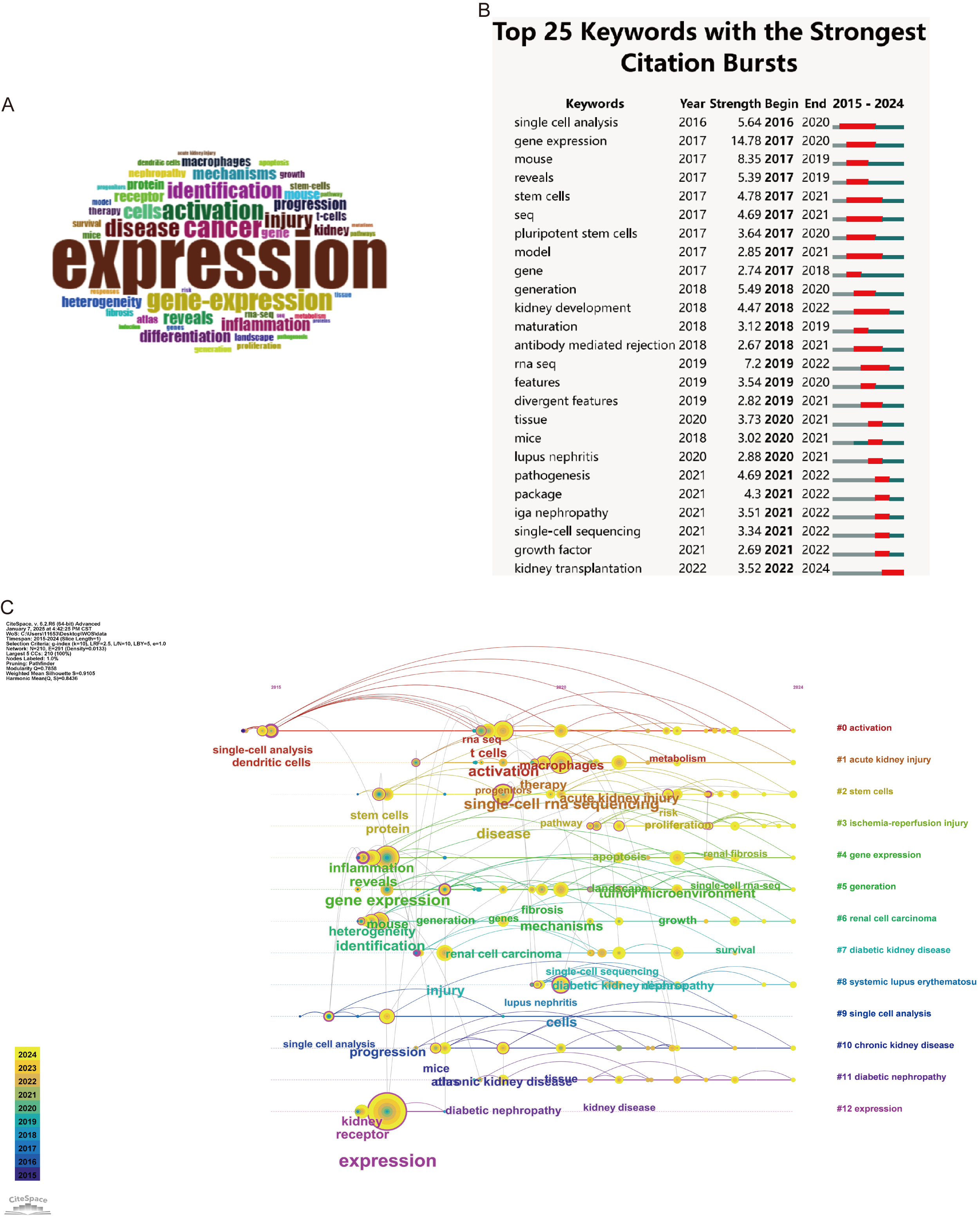

Supplement: Figure1-8.zip [file IRNF_A_2521457_SM2502.zip › Figure1-8/Figure6.tif]

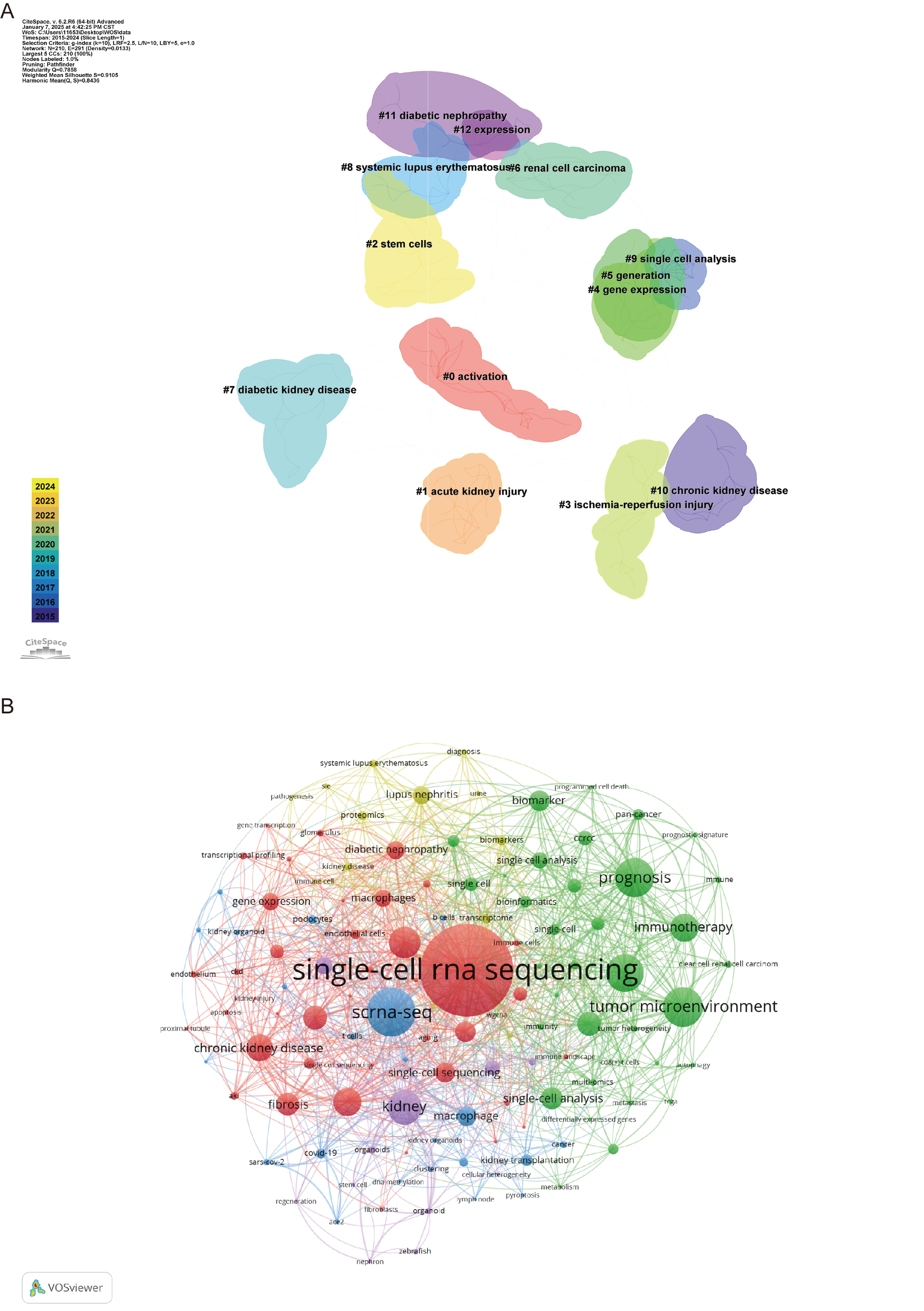

Supplement: Figure1-8.zip [file IRNF_A_2521457_SM2502.zip › Figure1-8/Figure7.tif]

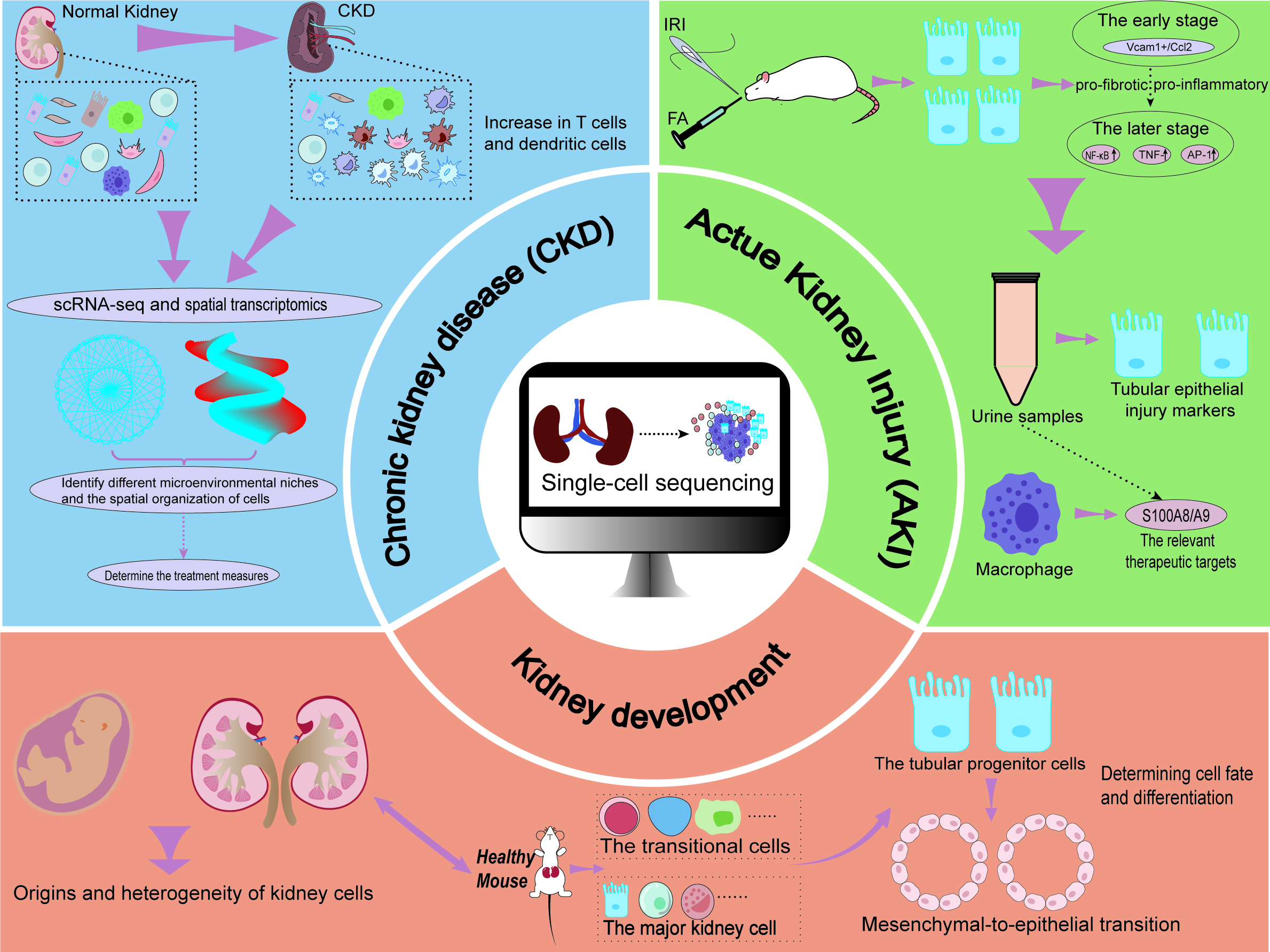

Supplement: Figure1-8.zip [file IRNF_A_2521457_SM2502.zip › Figure1-8/Figure8.tif]
